# Demotivated, but still attentive: Text disfluency does not affect mind-wandering and reading comprehension, but reduces motivation

**DOI:** 10.3758/s13423-025-02735-0

**Published:** 2025-08-07

**Authors:** Steffen Tietz, Marlene Müller, Jan Rummel, Lena Steindorf

**Affiliations:** 1 Department of Psychology, Psychology Institute, Heidelberg University, Hauptstraße 47-51, 69 117 Heidelberg, Germany; 2 TSG ResearchLab, Zuzenhausen, Germany

**Keywords:** Mind wandering, Reading, Disfluency, Text difficulty, Motivation, Reading comprehension

## Abstract

Studies on the relationship between text-processing difficulty, mind wandering, and reading comprehension achieved mixed results. Whereas most studies found mind-wandering frequency to be increased and reading comprehension to be decreased when text processing became more difficult, Faber et al. (*Psychonomic Bulletin and Review 24*(3), 914–919, 2017) reported an opposite effect when manipulating text difficulty via different font types (i.e., Arial vs. Comic Sans). This effect may reflect a potential of mildly disfluent fonts, such as Comic Sans, to introduce desirable difficulties during reading, thereby enhancing focus on the text. Strongly disfluent fonts, however, may contribute to the commonly observed disadvantages in text focus under conditions of increased text processing difficulty. To test this idea, we conducted a new study (*N* = 151, student sample) in which we manipulated disfluency in three levels (i.e., fluent, mildly disfluent, strongly disfluent) by using different font types, and compared mind-wandering frequency, reading comprehension, and reading motivation between conditions. The disfluency manipulation affected motivation but not mind wandering or reading comprehension. Additional Bayesian analyses strongly supported the null hypothesis for the latter two. These results suggest that the positive effects of reading disfluency may be less robust than previously assumed and that further research is needed to explore to which extent text-processing difficulty effects on mind wandering are reliant on sample and text characteristics.

## Introduction

The experience of one’s own thoughts drifting away from the here and now towards inner thoughts and feelings is a frequent phenomenon in everyday life. This experience has been studied by psychologists under the umbrella term “mind wandering”, a term that generally covers a wide range of phenomena, such as task-unrelated thoughts, stimulus-independent thoughts, unintentional thoughts, unguided thoughts, or daydreaming (Christoff et al., 2016; Irving et al., 2020; Seli et al., 2018). For the present work, however, we adopt the narrower definition of mind wandering as task-unrelated thought, that is, thoughts that occur while performing a task but are disengaged from this very task (Smallwood & Schooler, 2006). Such thoughts have been shown to occur relatively frequently during everyday activities (Kane et al., 2017). It is thus not surprising that mind wandering is also quite prevalent during reading (D'Mello & Mills, 2021). Importantly, mindless reading comes at a cost, as it is negatively associated with reading comprehension. A meta-analysis by Bonifacci et al. (2023) shows that this relationship is substantial (*r* =  − 0.21). It is thus important to understand which factors influence the likelihood with which mindless reading occurs.

Generally, a higher motivation to read a given text (e.g., due to a higher topic interest) has been shown to go along with fewer instances of mind-wandering (Krawietz et al., 2012; Soemer & Schiefele, 2019; Soemer et al., 2023, 2024; Steindorf et al., 2023; Unsworth & McMillan, 2013). Previous research further suggests that mind wandering is affected by the demands imposed by the reading task. Most studies found mind wandering to occur more frequently when text difficulty was increased by extending word or sentence length (Feng et al., 2013; Mills et al., 2015, 2017; Kahmann et al., 2021; Soemer & Schiefele, 2019; Soemer et al., 2019; Smallwood & Schooler, 2006; but see Forrin et al., 2019, Experiment 1a; Fulmer et al., 2015, for moderating effects). A similar pattern was observed in a recent study which imposed visual noise during reading resulting in increased mind wandering and reduced reading comprehension (Steindorf et al., 2023).

Most likely, reading motivation and reading demands are not independent of each other, as reading motivation seems to drop when text or perceptual demands during reading are increased (Soemer & Schiefele, 2019; Steindorf et al., 2023). This decrease in motivation may occur because higher demands might make reading feel more effortful and less rewarding, potentially leading to frustration or disengagement. Interestingly, a recent meta-analysis indicates that neither motivation nor text difficulty moderate the mind-wandering–reading-comprehension relationship, hinting towards these experimental effects being more fragile and dependent on moderating variables than assumed (Bonifacci et al., 2023).

However, there is a notable exception from the larger bulk of literature showing a positive relationship between reading demands and mind-wandering frequency. Faber et al. (2017) observed *fewer* instances of mind wandering when the same to-be-read text was presented in a less fluent font (Comic Sans, gray) compared to a more fluent font (Arial, black). This is an important finding because if text disfluency does indeed result in fewer mind-wandering instances, it could qualify as a *desirable* difficulty during reading (cf. McDaniel & Einstein, 2005). Given the negative association between mind wandering and reading comprehension often observed (Bonifacci et al., 2023), this finding is of great interest for theoretical but also applied purposes.

Interestingly, despite its preventative effect on mind wandering, the font-type manipulation had no effect on reading comprehension in the Faber et al. (2017) study, challenging the idea that text disfluency is desirable. The authors also did not find evidence for any motivational changes due to the disfluency manipulation.

At first glance, Faber et al.’s results seem to be somewhat contradictory to the bulk of previous studies showing mind wandering to increase when reading becomes more difficult. The disfluency manipulation via font types, however, is also quite different from the text difficulty manipulations in most other studies. That is, the disfluency manipulation targets text processing at an early perceptional stage.

According to the construction-integration model (e.g., Kintsch, 2005), reading comprehension involves three hierarchical levels of processing. On the first level, the perceptual information from the written text needs to be extracted to find its lexical meaning. On the second level, words need to be organized in propositions to find their semantic meaning. On the third level, the propositions need to be related to the reader’s world knowledge to understand their meaning in the particular situation. While the first two processing levels require little attention for literate readers, third-level processing regularly requires decent amounts of attention. It is thus not surprising, that people with a high propensity to mind wander show worse reading comprehension performance (McVay & Kane, 2011).

One explanation for why mind wandering regularly increases with increasing text difficulty is that situational mental model-building during reading (i.e., third-level processing) is hampered when the to-be-read text becomes overly difficult to understand (Smallwood et al., 2008). The disfluency manipulation by Faber et al. (2017), which targets perceptual processing of the text rather than text understanding, however, may foster an on-task focus in a bottom-up fashion and at an earlier level of text processing (Kintsch, 2005), so that mind wandering during reading will decrease with increased perceptual demands as long as readers’ abilities are not exceeded (cf. D'Mello & Mills, 2021). This might explain why Steindorf et al. (2023) found a mind-wandering increase rather than decrease when imposing perceptual background noise on a to-be-read text. Indeed, manipulation check data which suggest that Steindorf et al.’s (2023) participants felt impaired by the background noise whereas Faber et al.’s (2017) participants did not judge the reading of the less fluent font to be more difficult than reading of the fluent font, support this assumption.

In the present study, we aimed to replicate the preventative effect of text disfluency on mind wandering observed by Faber et al. (2017). In line with the considerations made above, we also added a third condition with a strongly disfluent font. Based on Faber et al.’s findings, we hypothesized that making the text slightly disfluent should impose “desirable difficulties” which make the task conveniently challenging, thereby reducing mind wandering. However, making the text very disfluent should impose “undesired difficulties” which render the task inconvenient to perform, thereby increasing mind wandering as observed in the Steindorf et al. (2023) study. That is, we expected a U-shaped relationship between the levels of disfluency and mind wandering. Motivational changes may accompany the hypothesized disfluency–mind-wandering relationship. Motivation to perform a task is known to be encouraged when task difficulty increases slightly but discouraged when it gets overly high (Yerkes & Dodson, 1908). Thus, although Faber et al. (2017) did not observe disfluency effects on reported motivation, we asked people to report their current motivation.

## Methods

Across the subsequent Methods and Results sections, we report how we determined our sample size and all data exclusions, manipulations, and measures in the study (Simmons et al., 2012). The present study was preregistered at the Open Science Framework (OSF) (https://osf.io/kqgbp/?view_only=043c74b9c0384db992bf4cc6a1b757fa).

### Participants and design

Data were collected from 11 November to 6 December 2022. To allow for stable parameter estimation within the intended multilevel modeling analyses, we aimed to collect valid data of *N* = 150, following recommendations by Maas and Hox (2005). We slightly oversampled to account for potential data exclusions. We collected *N* = 165 in total and applied the exclusion criteria from the preregistration. Data of *n* = 3 participants not meeting the preregistered participation criteria (German as their mother tongue and a minimum age of 18 years) were excluded, as well as data of *n* = 4 with unusual reading times (more than three standard deviations above or below the condition average), data of *n* = 3 participants who prematurely terminated the study due to technical problems, and data of *n* = 2 who stated after the experiment that they had not complied with the instructions. Additionally, we excluded data of *n* = 1 participant who had changed the background color of the text and data of *n* = 1 participant for whom our fluency manipulation was not displayed correctly due to a technical error.

The final sample consisted of *N* = 151 German native speakers with a mean age of 22.11 (*SD* = 3.58) years, of which *n* = 114 were female, *n* = 34 were male, and *n* = 3 did not identify as male or female; *n* = 142 of the final sample were students, *n* = 5 were former students, and *n* = 4 were non-students.

Text disfluency was manipulated between participants in three levels (fluent, mildly disfluent, strongly disfluent).

### Materials

#### Reading material and reading comprehension assessment

We used a German scientific article on evolution and change of the world from Wink (2016) as reading material. Because of its length, we shortened the article, so that the text comprised 2,780 words (127 sentences). Additionally, some spelling mistakes were corrected, and some odd terms were slightly adapted. As in the study by Faber et al. (2017), the text was presented on the screen sentence-by-sentence. Participants used the spacebar to proceed to the next sentence.

Reading comprehension was assessed via a single-choice test with 18 self-developed items and four response options per item. The reading material and the reading comprehension questions are provided on the OSF (https://osf.io/kqgbp/?view_only=043c74b9c0384db992bf4cc6a1b757fa).

#### Manipulation of disfluency

**Fig. 1:**
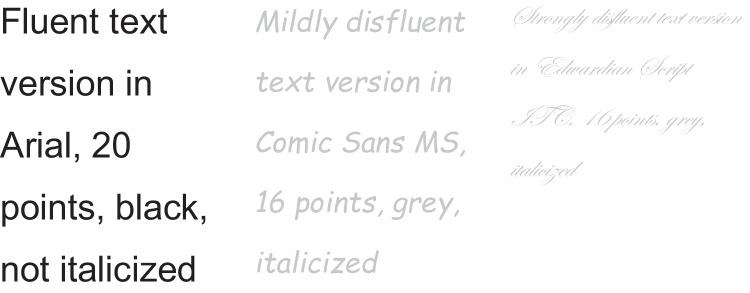
Visualization of the three disfluency conditions. From left to right: fluent, mildly disfluent, strongly disfluent

We replicated the disfluency manipulation (fluent, mildly disfluent) from Faber et al. (2017), and added a third condition which was intended to be more disfluent than the other two conditions. Specifically, we manipulated typeface, font size, font color, and whether the font was italicized or not. In the fluent condition, we used Arial, 20 points, black, not italicized, and in the mildly disfluent condition, we used Comic Sans MS, 16 points, gray, italicized (see Faber et al., 2017). In the strongly disfluent condition, we used Edwardian Script ITC, 16 points, gray, italicized. Examples of the three fonts are displayed in Fig. 1.

As a manipulation check, we used three self-developed items measuring perceived disfluency (which we intended to manipulate), perceived semantic difficulty (to differentiate our perceptual manipulation from a semantic manipulation), and general perceived reading difficulty. We also used the two items by Faber et al. (2017) measuring perceived text difficulty and perceived competence. All items are listed in Table 1.

**Table 1:** Post-reading manipulation check items

Variable	German	English translation
Perceived disfluency (self-developed item)	Die Darstellungsweise des Textes hat mir das Lesen erschwert	The presentation format of the text made it difficult to read
Perceived semantic difficulty (self-developed item)	Der Inhalt des Textes war schwer verständlich für mich	The content of the text was difficult to understand
General reading difficulty (self-developed item)	Alles in allem war es schwierig für mich, den Text zu lesen	All in all, I found it difficult to read the text
Perceived text difficulty (Faber et al., 2017)	Ich glaube, das Leseniveau des Textes war sehr schwierig	I believe the reading level of the text was very difficult
Perceived competence (Faber et al., 2017)	Ich denke, ich habe den Text sehr gut verstanden	I think I understood the text very well

All items were scaled from 1 (stimme überhaupt nicht zu/strongly disagree) to 6 (stimme vollkommen zu/strongly agree)

#### Mind-wandering conceptualization and assessment

Mind wandering was defined as the occurrence of task-unrelated thoughts. This definition was explained to the participants and described as a natural phenomenon that happens to everyone from time to time (cf. Faber et al., 2017). Mind-wandering was measured via thought-probes (Weinstein, 2017). In line with Faber et al. (2017) probes asked participants: “Were you just mind wandering? “ (response options: “yes” or “no”). In total, there were 14 probes which occurred every 155–280 words (*M* = 196.29, *SD* = 37.42), always at the end of a sentence. The number of words and sentences between probes varied to make sure that participants could not predict the appearance of the probes.

After reading, participants were additionally asked to retrospectively categorize the entirety of their thoughts during the reading phase into the two thought-probe response categories (on-task and off-task) using percentage scores.

#### Motivation assessment

Directly after each thought probe, the current motivation was measured using motivation probes (probe: “I have just been very motivated to read the text”; response options: rating score from 1 = “strongly disagree” to 6 = “strongly agree”).

Retrospective motivation considering the complete reading phase was assessed with four items on a 6-point scale measuring effort, interest, perceived value and general motivation (see Table 2). The first three of these items shown in Table 2 have been used by Faber et al. (2017) and were published by Phillips et al. (2016). We added one further item that was similar to our motivation probes to also measure reading motivation retrospectively.

**Table 2:** Retrospective assessment of effort, interest, perceived value, and motivation

	German	English translation
Effort	Ich habe viel Arbeit hineingesteckt	I put a lot of effort into this
Interest	Ich würde diese Aktivität als sehr interessant beschreiben	I would describe this activity as very interesting
Perceived value	Ich glaube, diese Aktivität könnte vorteilhaft für mich sein	I believe doing this activity could be beneficial to me
Reading motivation	Ich war sehr motiviert, den Text zu lesen	I was very motivated to read the text

All items should be rated from 1 (stimme überhaupt nicht zu/strongly disagree) to 6 (stimme vollkommen zu/strongly agree). The first three items were adopted from Phillips et al. (2016)

#### Compliance and technical issues questions

As preregistered, to exclude non-compliant participants and participants with serious technical problems, we asked participants after the experiment the following yes/no questions: “Please be honest, the answer does not affect the reimbursement: Did you actually read the text, and did you seriously attempt to answer the questions?” and “Did you experience any technical difficulties while reading? For example, did you have the feeling that after pressing the space bar the next sentence was occasionally displayed with a clear delay?” Participants responding “yes” to thee first question were excluded and participants answering “yes” to the second one were asked to specify the problems and were excluded when problems were likely to have hampered the experimental manipulation (e.g., disfluent text presentation due to network problems).

### Procedure

Participants were invited to a laboratory at the Department of Psychology at Heidelberg University and were informed about the terms of conditions for participation. After giving informed consent, participants started the experiment, which was implemented using the Qualtrics software (Qualtrics, 2022). First, participants provided basic demographic information before receiving task instructions. Participants were randomly assigned to one of the three experimental conditions and read the corresponding text version (fluent, mildly disfluent, strongly disfluent). The text presentation was interrupted 14 times by a thought probe and a subsequent motivation probe. After reading, participants performed the reading comprehension test and answered the retrospective questions about mind wandering and motivation during reading as well as the compliance and technical issues questions before being debriefed and dismissed.

## Results

The analyses reported in this section were executed in accordance with the preregistration if not explicitly indicated otherwise. Significance tests were calculated one-tailed whenever testing preregistered directed hypotheses and two-tailed in all other cases.

### Manipulation check

We tested whether perceived perceptual disfluency, semantic difficulty, and retrospectively assessed reading difficulty were affected by our disfluency manipulation. For this purpose, we conducted ANOVAs with disfluency levels as a between-participants factor for all three variables. Significant results were followed up with post hoc comparisons to locate the significant condition differences. For perceptual disfluency, we slightly adapted this preregistered procedure due to a violated assumption of variance homogeneity (indicated by a significant Levene test) using a Welch-ANOVA and a Games-Howell-Test instead.

Descriptive values for the three manipulation check items are displayed in Table 3. As expected, we found no significant difference in semantic difficulty (*F*(2, 148) = 1.92, *p* = 0.150), but significant differences in general difficulty (*F*(2, 148) = 50.76, *p* < 0.001) and in perceptual disfluency (*F*(2, 85.784) = 133.97, *p* < 0.001).

**Table 3:** Manipulation check for level of disfluency

	Fluent		Mildly disfluent		Strongly disfluent	
	*M*	*SD*	*M*	*SD*	*M*	*SD*
Perceived semantic difficulty	2.43	0.96	2.55	1.10	2.83	1.14
Perceived general difficulty	2.33	1.03	2.63	1.13	4.33	1.04
Perceived disfluency	2.69	1.48	3.45	1.37	5.75	0.60
Perceived reading difficulty (Faber et al., 2017)	2.83	1.02	2.65	1.13	2.92	1.15
Perceived competence (Faber et al., 2017)	4.30	0.86	4.20	0.93	4.02	1.04

All ratings ranged from 1 to 6. Higher numbers represent higher expressions in the measured variable

Post hoc comparisons revealed the expected significant increases in perceptual disfluency and general difficulty with increasing levels of disfluency. This increase in general difficulty was only trending between the fluent and the mildly disfluent condition (*p* = 0.078) but significant between the mildly and the strongly disfluent condition (*p* < 0.001). For perceptual disfluency, the increase from the fluent to the mildly disfluent condition was significant (*p* = 0.011) and so was the one from the mildly to the strongly disfluent condition (*p* < 0.001), indicating that disfluency manipulation was successful.

In addition to the preregistered manipulation check, we analyzed the retrospective difficulty and competence estimates using adopted versions of the items by Faber et al. (2017). Descriptive values are shown in Table 3. In line with Faber et al., we found no significant differences between disfluency levels in perceived difficulty (*F*(2, 148) = 0.74, *p* = 0.481) and in perceived competence (*F*(2, 148) = 1.11, *p* = 0.334).

#### Disfluency effects on mind wandering

The average mind-wandering rate measured online with the 14 mind-wandering probes was *M* = 35.15% (*SD* = 47.75%) and the retrospectively reported overall rate was very similar, *M* = 35.82% (*SD* = 23.05%). Disfluency condition means and standard deviations are displayed in Table 4. To investigate the relationship between mind-wandering and disfluency levels, a generalized linear mixed-effects model with mind wandering (binary coded with 0 for on task and 1 for mind-wandering responses) as criterion was specified. We used a logic-link function to relate the binary criterion to the predictor variables. Random intercepts were estimated for each participant and time-on-task was included as fixed effect. As stated in the preregistration, for the disfluency manipulation we specified contrasts that represented our hypotheses, namely a linear contrast (−1: fluent, 0: mildly disfluent, 1: strongly disfluent) and a quadratic contrast (1: fluent, −2: mildly disfluent, 1: strongly disfluent). Contrary to our hypothesis, including level of disfluency as an additional predictor did not significantly improve the model (*p* = 0.569), indicating that level of disfluency had no substantial effect on mind wandering (for the model results, see Table 5). Notably, using an ANOVA approach to analyze the mind-wandering data yielded the same results.

**Table 4:** Descriptive values for mind wandering, reading comprehension, and motivation depending on disfluency levels

	Fluent		Mildly disfluent		Strongly disfluent	
	*M*	*SD*	*M*	*SD*	*M*	*SD*
Mind-wandering (online)	33.20%	47.12%	35.13%	47.77%	37.35%	48.41%
Mind-wandering (retrospective)	32.04%	24.90%	36.71%	21.61%	39.17%	22.16%
Reading comprehension	56.38%	49.62%	51.93%	49.99%	50.35%	50.03%
Motivation (online)	4.32	1.13	3.90	1.16	3.57	1.28
Effort (retrospective)	3.24	0.93	3.06	1.01	3.60	0.92
Interest (retrospective)	3.94	1.20	3.20	1.17	3.15	1.24
Perceived Value (retrospective)	4.22	1.06	3.53	1.32	3.48	1.30
Motivation (retrospective)	4.11	1.24	3.47	1.06	3.27	1.14

Mind wandering (online): Mean of reported mind wandering over all 14 thought probes, ranging from 0 to 100%. Mind wandering (retrospective): Retrospective estimates regarding which percentage of time was spent on mind wandering during reading. Reading comprehension: Mean percentage of correct answered reading comprehension questions. Motivation (online): Mean over all 14 motivation probes, ranging from 1 to 6, with higher numbers representing higher motivation. Effort, Interest, Perceived Value, Motivation (retrospective): Retrospective estimates of the respective variables on a scale ranging from 1 to 6

**Table 5:** Model results for the predictions of mind-wandering, reading comprehension, and motivation

	*b*	*SE*_*b*_	95% CI	*p*
**Mind wandering**				
Intercept	−0.81	0.12	[−1.04, −0.58]	< 0.001
Linear contrast	0.19	0.18	[−0.16, 0.55]	0.286
Quadratic contrast	0.02	0.31	[−0.60, 0.64]	0.947
Time on task	0.02	0.01	[−0.01, 0.04]	0.132
**Mind wandering (including reading time)**				
Intercept	−0.81	0.12	[−1.04, −0.58]	< 0.001
Linear contrast	0.20	0.19	[−0.17, 0.57]	0.286
Quadratic contrast	0.04	0.33	[−0.61, 0.68]	0.914
Time on task	0.02	0.01	[−0.01, 0.04]	0.132
Reading time	−0.01	0.08	[−0.17, 0.14]	0.864
**Mind wandering (including reading comprehension)**				
Intercept	−0.82	0.12	[−1.04, −0.59]	< 0.001
Linear contrast	0.10	0.18	[−0.25, 0.44]	0.576
Quadratic contrast	0.06	0.30	[−0.53, 0.66]	0.836
Time on task	0.02	0.01	[−0.01, 0.04]	0.132
Reading comprehension	−0.26	0.07	[−0.40, −0.12]	< 0.001
**Mind wandering (including text halves)**				
Intercept	−0.87	0.13	[−1.13, −0.61]	< 0.001
Linear contrast	0.19	0.18	[−0.16, 0.55]	0.286
Quadratic contrast	0.02	0.32	[−0.60, 0.64]	0.947
Time on task	0.04	0.02	[−0.01, 0.09]	0.108
Text halves	−0.19	0.19	[−0.57, 0.19]	0.321
**Reading comprehension**				
Intercept	0.14	0.18	[−0.22, 0.50]	0.444
Linear contrast	−0.29	0.16	[−0.61, 0.03]	0.075
Quadratic contrast	0.13	0.29	[−0.42, 0.69]	0.639
**Motivation**				
Intercept	3.86	0.08	[3.71, 4.02]	< 0.001
Linear contrast	−0.75	0.17	[−1.08, −0.41]	< 0.001
Quadratic contrast	0.09	0.30	[−0.49, 0.68]	0.760
Time on task	0.01	0.00	[−0.00, 0.02]	0.063

Mind wandering and motivation were assessed with online probes during the reading phase. Reading comprehension was assessed after the reading phase. Linear contrasts test for a linear increase of the dependent variable with increasing level of disfluency. Quadratic contrasts test for a difference on the respective dependent variable between the mildly disfluent condition and to the other two conditions (fluent and strongly disfluent). Time-on-task tests for systematic changes with probe occurrence (from 1 to 14). Text halves tests whether the mind wandering effects changed from the first (probes 1 to 7) to the second (probes 8 to 14) text half

We conducted additional Bayesian analysis for this effect, which was not preregistered but seemed useful for the interpretation of the null finding. Using brms-package and Bayes-Factor-package in RStudio, we found a Bayes factor of *BF*_*01*_ = 18.43 in favor of a null effect of the disfluency manipulation on mind wandering, indicating strong evidence for this null effect according to common interpretations of Bayes factors (Jarosz & Wiley, 2014).^1^

In addition to the preregistered analyses, we tested for differences in retrospectively assessed mind-wandering frequency between the three levels of disfluency and also found no significant differences (*F*(2, 148) = 1.27, *p* = 0.283).

In response to comments from two reviewers, we exploratively tested for the potential role of reading abilities in explaining the null effect of disfluency on mind wandering and also for changes over time. For this purpose, we first added reading time as an additional predictor to the original mind-wandering model (see Table 5). Allowing for an interaction between reading time and our disfluency condition did not significantly improve this model (*p* = *0*.655), and a Bayes factor of *BF*_*01*_ = 83.41 in favor of the null hypothesis showed very strong evidence that there was indeed no interaction effect between disfluency and reading time on mind wandering. Second, we added the sum of correctly answered reading comprehension questions to the original mind-wandering model (see Table 5). Allowing for an interaction also did not significantly improve this model (*p* = 0.715), and a Bayes factor of *BF*_*01*_ = 136.01 in favor of the null hypothesis showed decisive evidence that there was indeed no interaction effect between disfluency and reading comprehension on mind-wandering. Taken together, we did not find any hints in our explorative analyses that reading abilities measured by reading times or reading comprehension interacted with disfluency in a way that might explain the null effect of disfluency on mind-wandering.

Finally, we added the text halves (first vs. second) as a predictor to the original mind-wandering model to test whether disfluency effects would change over time (see Table 5). Allowing for an interaction between text halves and disfluency did not improve the model fit (*p* = 0.409), and a Bayes factor of *BF*_*01*_ = 48.77 in favor of the null hypothesis showed very strong evidence that there was no interaction effect between disfluency and text half on mind-wandering.

#### Disfluency effects on reading comprehension

The average reading comprehension performance across all three conditions was *M* = 53.02% (*SD* = 49.92%). Disfluency condition means are displayed in Table 4. To investigate the relationship between reading comprehension and level of disfluency, a generalized linear mixed-effects model with reading comprehension (binary coded with “0” for incorrect and “1” for correct responses) as criterion was specified. We used a logic link function to relate the binary criterion to the predictor variables. Crossed-random intercepts were estimated for each participant and each question. We included the level of disfluency as additional fixed effect by specifying a linear and a quadratic contrast.^2^ Contrary to our expectations, including level of disfluency as additional fixed effect did not significantly improve the model (*p* = 0.182) indicating that level of disfluency had no effect on reading comprehension. The Bayes factor in favor of this null effect was *BF*_*01*_ = 7.39, showing substantial evidence for the null hypothesis.

#### Disfluency effects on motivation

The average motivation score across all three conditions was *M* = 3.94 (*SD* = 1.23). Disfluency condition means are displayed in Table 4. To investigate the relationship between the current motivation and level of disfluency, a mixed-effects model with motivation (variable ranging from “1” = “strongly disagree” to “6” = “strongly agree”) as criterion was specified. Random intercepts were estimated for each participant. Time on task was included as a fixed effect. We included the level of disfluency as additional fixed effect by specifying a linear and a quadratic contrast.^3^ As expected, adding level of disfluency as a further fixed effect improved the model significantly (*p* < 0.001), and as expected we found the linear contrast to be significant, indicating lower motivation with increasing level of disfluency (see Table 5). The Bayes factor in favor of the alternative hypothesis was *BF*_*10*_ = 265.14, showing decisive evidence for the influence of the level of disfluency on motivation.

In addition to the preregistered analyses, we tested for differences between the fluency conditions in the retrospective questionnaire (see Table 4). We found significant differences in effort (*F*(2, 148) = 4.09, *p* = 0.019), interest (*F*(2, 148) = 7.10, *p* = 0.001), perceived task value (*F*(2, 148) = 5.93, *p* = 0.003), and retrospectively assessed motivation (*F*(2, 148) = 7.49, *p* < 0.001). Post hoc comparisons revealed significant differences in effort between the mildly and the strongly disfluent condition (*p* = 0.006), but not between the fluent and the mildly disfluent condition (*p* = 0.341). Concerning value, interest, and general motivation, there were significant differences between the fluent and the mildly disfluent condition (*p* = 0.002; *p* = 0.005; *p* = 0.006), but not between the mildly and the strongly disfluent condition (*p* = 0.812; *p* = 0.837; *p* = 0.398).

Further preregistered analyses concerning potential moderating effects of disfluency strength on the relationship between mind wandering and reading comprehension are provided on the OSF (https://osf.io/kqgbp/?view_only=043c74b9c0384db992bf4cc6a1b757fa). We decided to report these additional analyses, which were not central to our main hypothesis, as supplementary analyses so as not to distract the readers with these inconclusive results.

## Discussion

The aim of this study was to replicate and extend Faber et al.’s (2017) results of decreased mind wandering under increased text-processing difficulties. We assumed that the perceptual text-difficulty manipulation of using an uncommon font type used by Faber et al. (2017) was subtle enough not to interfere with cognitive processing during reading (while eventually imposing a desirable reading difficulty). In contrast, other manipulations such as adding visual background noise (Steindorf et al., 2023) or increasing paragraph lengths (Mills et al., 2015) may interfere with reading at the perceptual, propositional, or situational processing level. To test this assumption, we replicated Faber et al.’s design as closely as possible with our German materials and added a third experimental condition. In this condition, we intended to turn the supposedly desirable difficulties of a moderately disfluent text into undesired difficulties of a strongly disfluent text by using a quite inconveniently to-be-read font.

Our manipulation-check clearly demonstrated that we successfully manipulated the perceptual difficulties through the disfluency manipulation. However, other than hypothesized, we did not find any effect of the disfluency manipulation on either mind-wandering frequency or reading comprehension. Additional Bayesian analyses showed that there was good evidence that there was indeed no effect of the disfluency manipulation on either variable.

The reading comprehension results align with the results of Faber et al. (2017). Our results even suggest that further increasing reading disfluency still has no effect on reading comprehension. The null effect on mind wandering we observed is, however, at odds with Faber et al.’s results. This is unexpected because we used the same disfluency manipulation as well as quite similar materials. Our German text was only slightly longer (2,780 words) than the English one (1,491 words) used by Faber et al. (2017) and, correspondingly, we implemented a few more thought probes (14 vs. 9). However, when we included text half as an additional predictor in our mind-wandering analyses it did not interact with the disfluency manipulation. So, there is no evidence that the length of our text was responsible for the divergent findings. Further, as preregistered, instead of a Mann–Whitney *U* test, we used multi-level models to analyze the mind-wandering data. We deemed our approach to be better-suited for the three-fluency-levels manipulation and the dichotomously assessed dependent variable. Furthermore, it has a higher statistical power, as it allows us to control for time-on-task effects (via detrending) and individual differences in the responsiveness to the fluency manipulation (via including random intercepts for participants). All these methodological differences between studies, however, are unlikely to be responsible for the different results.

One potential reason why we did not replicate Faber et al.’s (2017) results in the present study might be differences in the samples obtained in the two studies. While Faber et al. (2017) collected data online from Amazon Mechanical Turk (MTurk) workers, we collected data in a laboratory setting, primarily from a student sample. It is possible that the online setting and/or the more cognitively diverse MTurk sample contributed to the divergent results, as online settings (Cotton et al., 2023) and people with low cognitive abilities (McVay & Kane, 2011) are particularly prone to mind wandering. Another noteworthy difference is that, according to the Flesch index (Flesch, 1948), the text we used qualifies as a difficult text for high school students (which seems appropriate for a student sample), whereas the text used by Faber et al. (2017) qualifies as a moderately difficult text (which, again, seems appropriate for a general public sample). The calculation of Flesch indices is documented in the OSF repository.

Taken together, further research is needed to better understand the boundary conditions for the disfluency effects on mind wandering observed by Faber et al. (2017). In this regard it will be crucial to consider differences in text difficulty and cognitive and reading abilities in a more fine-grained fashion than performed in the present experimental replication approach.

An interesting side note is that we found the motivation to drop significantly when the text was presented in a strongly disfluent font as compared to a fluent or mildly disfluent font. Typically, lower motivation has been shown to go along with more frequent mind wandering and poorer reading comprehension (Soemer & Schiefele, 2019; Steindorf et al., 2023), a pattern that was not observed in the present study. One reason might be that even the strongly disfluent font was still easy enough to read so that it did not hamper processing during reading for our student sample. On a theoretical level, these results are certainly interesting as they show that a reduced motivation to read a text does not always result in increased mind wandering and decreased reading comprehension.

## Conclusion

In the present study, we found that reading disfluency did not affect mind wandering or reading comprehension. Nevertheless, a strongly disfluent font reduced reading motivation. The results do not replicate previous findings by Faber et al. (2017) and speak against the idea that reading disfluency and mind wandering are related in a U-shaped fashion. Despite some open questions, the present findings contribute to a better understanding of the factors influencing mind wandering during reading.

## Data Availability

Data and other materials are available on the OSF via the following link: https://osf.io/kqgbp/?view_only=043c74b9c0384db992bf4cc6a1b757fa.

## Footnotes

1 To obtain a stable Bayes factor estimate with the R-package brms, we estimated our models with an increased number of iterations (100,000 across four chains). When doing so, there were only minor variations in the decimal places of the estimated Bayes factors when running the analyses repeatedly.

2 Please note that there was a contrast coding error in our preregistration so that the second contrast was not orthogonal to the first one, which we corrected for the present analyses.

3 Please note that there was a contrast coding error in our preregistration so that the second contrast was not orthogonal to the first one, which we corrected for the present analyses.

## Code availability

The analysis code is available on the OSF via the following URL: https://osf.io/kqgbp/?view_only=043c74b9c0384db992bf4cc6a1b757fa.
